# Pain Trajectories in Knee Osteoarthritis—A Systematic Review and Best Evidence Synthesis on Pain Predictors

**DOI:** 10.3390/jcm9092828

**Published:** 2020-09-01

**Authors:** Davide Previtali, Luca Andriolo, Giorgio Di Laura Frattura, Angelo Boffa, Christian Candrian, Stefano Zaffagnini, Giuseppe Filardo

**Affiliations:** 1 Orthopaedic and Traumatology Unit, Ospedale Regionale di Lugano, EOC, 6900 Lugano, Switzerland; davide.previtali@eoc.ch (D.P.); giorgiodilaura@gmail.com (G.D.L.F.); christian.candrian@eoc.ch (C.C.); ortho@gfilardo.com (G.F.); 2 Clinica Ortopedica e Traumatologica 2, IRCCS Istituto Ortopedico Rizzoli, 40136 Bologna, Italy; lucas.andriolo@gmail.com (L.A.); stefano.zaffagnini@unibo.it (S.Z.); 3 Facoltà di Scienze Biomediche, USI-Università della Svizzera Italiana, 6900 Lugano, Switzerland; 4 Applied and Translational Research (ATR) Center, IRCCS Istituto Ortopedico Rizzoli, 40136 Bologna, Italy

**Keywords:** knee, osteoarthritis, pain, trajectories, predictors, evolution

## Abstract

Different profiles of pain progression have been reported in patients with knee osteoarthritis (OA), but the determinants of this heterogeneity are still to be sought. The aim of this systematic review was to analyze all studies providing information about knee OA pain trajectories to delineate, according to patients’ characteristics, an evidence-based evolution pattern of this disabling disease, which is key for a more personalized and effective management of knee OA. A literature search was performed on PubMed, Web of Science, Cochrane Library, and grey literature databases. The Cochrane Collaboration’s tool for assessing risk of bias was used, and a best-evidence synthesis was performed to define the predictors of pain evolution. Seven articles on 7747 patients affected by knee OA (mainly early/moderate) were included. Daily knee OA pain trajectories were unstable in almost half of the patients. In the mid-term, knee OA had a steady pain trajectory in 85% of the patients, 8% experienced pain reduction, while 7% experienced pain worsening. Low education, comorbidities, and depression were patient-related predictors of severe/worsening knee OA pain. Conversely, age, alcohol, smoking, pain coping strategies, and medications were unrelated to pain evolution. Conflicting/no evidence was found for all joint-related factors, such as baseline radiographic severity.

## 1. Introduction

Knee osteoarthritis (OA) is, according to the World Health Organization (WHO), the eleventh leading cause of long-term disability worldwide [1]. Knee OA was traditionally perceived as a multifactorial progressing disease with invariably worsening symptoms, but, although this feature is evident in the long-term, symptoms progression is less clearly defined in the short- and mid-term follow-up [2,3]. Even though knee OA is characterized by plenty of hallmarks, pain is the main symptom of knee OA and it is the main target of treatment for this condition [4,5]. Nonetheless, little is known about the evolution of knee OA-related pain, which results in difficulties in predicting patient prognosis and properly evaluating the effectiveness of the administered therapy [6]. Countless treatments have been proposed, from rehabilitation protocols to surgery, to address OA-related functional limitations, but literature results are often inconclusive and heterogeneous [7,8,9,10]. In fact, each strategy is applied to the broad category of OA patients, without being able to distinguish from patients with different symptom patterns and evolution. This impairs the possibility for a personalized approach to effectively manage OA patients. In this light, the identification of specific OA patterns could help to better target rehabilitation protocols and conservative procedures, allowing to postpone the need for more invasive surgeries in responsive patients, while leaving surgery as an option for patients where less benefit is expected from less invasive strategies.

Thus, recent research efforts have been made to better characterize the different types of pain in patients with knee OA. In particular, different profiles of pain progression have been reported, but the determinants of this heterogeneity are still to be sought [11]. A better knowledge of the disease progression and the identification of the characteristics of patients more prone to develop a fast-progressive or more symptomatic form of knee OA is therefore needed. This could help general practitioners and specialists facing OA to predict the evolution of the disease and patient prognosis in order to improve expectations and management of knee OA patients [12,13]. This issue also plays a key role in the scientific research field, considering the importance of pain progression in the evaluation of the effectiveness of treatments, which should be compared to the normal disease progression pattern [14]. The identification of a clinically relevant endpoint is particularly important for promising new drugs, targeting the early phases of OA and aimed at modifying the progression of the disease [7,15,16]. Recently, the opportunity to follow-up communities of patients with knee OA made it possible to document the evolution of this disease and its symptoms [17]. Moreover, statistical models, able to identify trajectories by grouping patients with a common clinical disease progression, are increasingly used to classify and describe the evolution of symptoms over time [18,19].

The aim of this systematic review was to analyze all studies providing information about pain trajectories in knee OA patients, in order to delineate the evolution patterns of this disabling disease. Furthermore, a best evidence synthesis was performed to provide a characterization of patients with different pain progression, in order to identify the predictors of specific pain trajectories in patients with knee OA.

## 2. Experimental Section

### 2.1. Protocol and Registration

Methods of the analysis and inclusion criteria were specified in advance and documented in a protocol which was preregistered on PROSPERO (CRD42019119065). As compared to the previously published protocol of this systematic review, which initially also considered the trajectory of pain after total knee arthroplasty (TKA), this systematic review was then focused only on pain in patients with knee OA.

### 2.2. Eligibility Criteria

The eligibility criteria are reported in Table 1.

### 2.3. Information Sources

A systematic literature search was performed on 25 June 2020 on PubMed, Web of Science, Cochrane library, and grey literature databases (isrctn.org, clinicaltrials.gov, greylit.org, and opengrey.eu).

### 2.4. Search

The search was performed using the string: (“pain trajectories” OR “pain trajectory” OR “pain curve” OR “pain curves”). No limits were used.

### 2.5. Study Selection

At first, extracted records were screened for duplicates’ removal and subsequently selected applying the eligibility criteria previously reported (Table 1). A first screening was performed by title and abstract, whereas a full-text reading evaluation was necessary when not enough information was available from the abstract. The Preferred Reporting Items for Systematic Reviews and Meta-analysis (PRISMA) guidelines were used [20] (Supplementary Table S1). Two authors (D.P. and L.A.) independently performed the article selection process with disagreement solved by consensus or by the intervention of a third author (G.D.L.F.).

### 2.6. Data Collection Process

Relevant information from eligible articles was independently collected by two authors (D.P. and L.A.), using a previously structured table according to Cochrane [21].

### 2.7. Data Items

Information on study methodology concerned: level of evidence, study design, pain score, method of pain trajectories assessment, inclusion/exclusion criteria, and origin of data. Data on the characteristics of the study population were also extracted: number of patients, follow-up, sex, age, race, body mass index (BMI), level of instruction, psychological profile (anxiety, depression), comorbidities, activity level, symptoms’ duration, functional scores, characteristics of pain and patient’s pain experience (pain coping strategies), perceived health status, use of medications, surgery rate, and radiological severity of knee OA.

### 2.8. Risk of Bias in Individual Studies

The Cochrane Collaboration’s tool for assessing the risk of bias of prognostic studies was used. This tool consisted of 8 questions: 2 questions about selection bias, 4 questions about information bias, and 2 questions about confounders. To be considered as having a low risk of bias, a study should satisfy at least 6 out of 8 criteria and at least 1 criterion for each risk of bias category (selection bias, information bias, confounding). Studies with a moderate risk of bias satisfy at least 5 out of 8 criteria and at least 1 criterion in 2 of the risk of bias categories. All other cases were considered as high risk of bias. The final decision was the result of the consensus between two of the authors (D.P. and G.D.L.F.).

### 2.9. Summary Measures

The distribution of patients in the different pain trajectories was computed and expressed as the proportion of the number of patients with a specific pain trajectory over the total number of patients. The range of proportions of patients belonging to that specific pain trajectory reported in the studies that identified such pain trajectory was also shown.

### 2.10. Synthesis of Results

A meta-analysis of proportions was attempted to quantify the prevalence of the different pain trajectories in patients with knee OA, as planned. Both fixed and random effects analyses were used, but the results of the random effect were preferred because of the heterogeneity of the included studies. Cochran’s Q statistic and I^2^ metric were used to quantify heterogeneity. The statistical analysis was performed with the packages meta (v4.9-7) and metafor (v2.1-0) in R Studio (v1.2.5019). However, the high heterogeneity of the retrieved data limited the statistical strength of the analysis and could not be resolved with the commonly accepted methods due to the low number of included studies. Thus, in order to avoid a misleading interpretation of the data, results were reported only as proportions, as explained in Section 2.9. The forest plots were reported in the Supplementary Figures S1–S13 for completeness [22].

### 2.11. Risk of Bias Across Studies

Finally, a best evidence synthesis was performed [23], following the indication of Van Tulder et al. [24] and the method of Eijgenraam et al. [25], in order to grade the evidence of factors (patient-related and joint-related) influencing pain evolution. The level of evidence was considered “strong” when results were provided by at least 2 studies with low risk of bias, with generally consistent findings in all studies (≥75% of the studies reported these findings). A “moderate” level of evidence was provided by 1 low risk of bias study and 2 or more moderate/high risk of bias studies, or by 2 or more moderate/high risk of bias studies, with generally consistent findings in all studies (≥75%). The evidence was considered “limited” when results were provided only by one low/moderate/high risk of bias studies with generally consistent findings (≥75%). If the findings were conflicting (<75% of the studies reported consistent findings), the evidence was also considered “conflicting”.

## 3. Results

### 3.1. Article Selection and Characteristics

**Figure 1:**
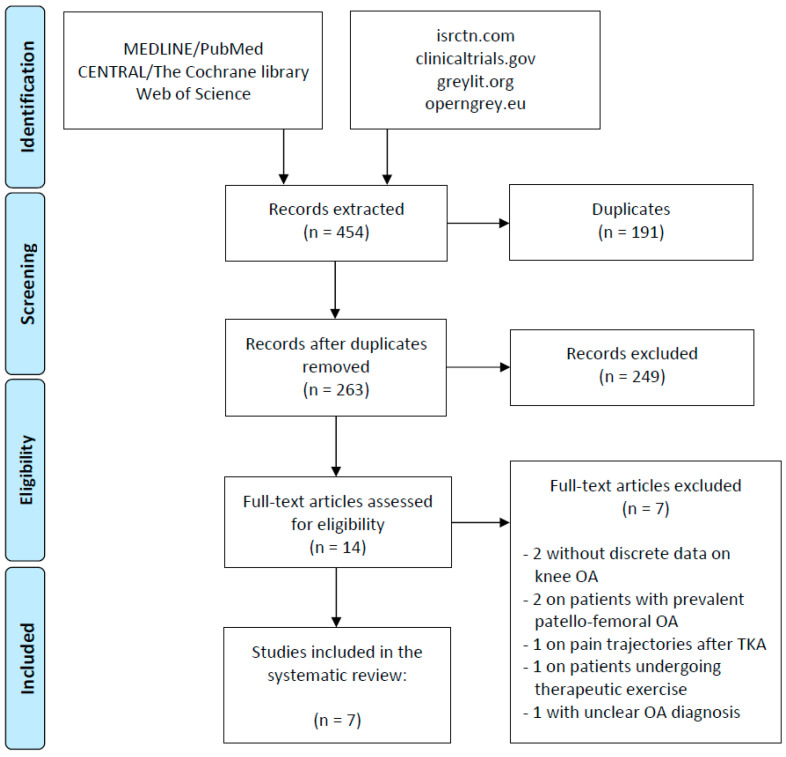
Preferred Reporting Items for Systematic Reviews and Meta-analysis (PRISMA) flowchart of the study selection process.

The initial search resulted in 454 titles from the included databases; among these, 191 were removed because they were duplicate references. Of the remaining 263, 249 were excluded according to the eligibility criteria, being studies without information on pain evolution or without discrete data for patients with knee OA, studies on patients treated with TKA or other surgical and non-surgical treatments, expert opinions (level of evidence V), reviews, systematic reviews and meta-analyses, or preclinical or ex vivo studies. Thus, 14 studies were assessed for eligibility, and out of these, seven full-text articles were excluded: two studies without discrete data on knee OA, two studies on patients with prevalent patello-femoral OA, one study on pain trajectories after TKA, one study on patients undergoing therapeutic exercise, and one study with unclear OA diagnosis. Thus, a total of seven articles, published from 2014 to 2018, were included in the systematic review (Figure 1). Data on knee OA patients were obtained from the Osteoarthritis Initiative (OAI) prospective cohort in three studies [3,26,27], from the CHECK prospective cohort in two studies [28,29], from the knee clinical assessment study (CAS-K) prospective cohort in one study [30], and from a cohort created by French general practitioners in one study [31].

These studies included 7747 patients, 7155 of which were examined until the last follow-up. Four studies included only one knee per patient (the most painful one), whereas three studies included both knees. Six studies had a follow-up of more than 5 years (range 5–8 years) [3,26,27,28,29,30], whereas one study documented pain trajectories on a daily basis for one month [31]. All studies were on cohorts where the majority of patients had an early knee OA (Kellgren–Lawrence ≤ 2 with knee pain and initial radiographic features of knee OA) [32,33]. The included studies and patients’ characteristics are reported in detail in Table 2.

### 3.2. Characteristics of Pain Trajectories in Knee OA Patients

In 5 out of 6 studies with a mid/long-term follow-up, the majority of patients reported a constant level of pain over the years. Among the 6 included studies, the proportion of patients included in a trajectory with constant pain was 85.4% (range 10–100%; I^2^ 97.6%). In particular, a constant minimal pain (less than 15% of the pain scale used) was detected in 43.5% of the patients (range 0–73%; I^2^ 99.7%), constant mild pain (level of pain between 15% and 30% on the pain scale used) was detected in 27.6% of the patients (range 0–67%; I^2^ 99.5%), constant moderate pain (level of pain between 30% and 50% on the pain scale used) was detected in 11.7% of the patients (range 0–42%; I^2^ 99.2%), and a constant severe pain (level of pain > 50% on the pain scale used) was detected in 2.6% of the patients (range 0–11%; I^2^ 92.4%).

The mean percentage of patients included in a trajectory with an increasing pain was 6.7% (range 0–31%; I^2^ 93.0%). In particular, no increasing trajectory in patients with initial minimal pain was reported in any of the included studies, an increasing mild pain was reported by 2.3% (range 0–28%; I^2^ 97.4%) of the patients, an increasing moderate pain was reported by 2.2% (0–26%; I^2^ 95.2%) of the patients, and an increasing severe pain was reported by 2.2% (range 0–27%; I^2^ 95.3%) of the patients.

The mean percentage of patients included in a trajectory with a decreasing pain was 7.9% (range 0–90%; I^2^ 98.2%). In particular, patients with decreasing mild pain were 3.4% (range 0–43%; I^2^ 95.6%), patients with decreasing moderate pain were 3.9% (range 0–32%; I^2^ 96.3%), patients with decreasing severe pain were 0.6% (range 0–15%; I^2^ 94.2%), whereas none of the studies detected a group of patients with decreasing minimal pain.

In the study that evaluated pain trajectories for one month, two different patterns of pain trajectories were studied: a stable pain trajectory (pain peaks during less than 25% of the month) characterized 59.5% of the patients, whereas an unstable pain trajectory (pain peaks during more than 50% of the month) characterized 40.5% of the patients. None of the patients reported a “stable with few peaks” pain trajectory (pain peaks during 25–50% of the month).

A meta-analysis of proportions was attempted to quantify the prevalence of the different pain trajectories in patients with knee OA. However, since the paucity and the high heterogeneity of the retrieved data limited the statistical strength and the relevance of the results of the meta-analysis, results were reported only as proportions, as explained in Section 2.9. The forest plots are reported in the Supplementary Figures S1–S13 for completeness.

### 3.3. Risk of Bias of the Included Studies

The risk of bias was judged to be low in 2 of the included studies, moderate in 4, and high in 1. Selection bias was almost surely absent in 3 of the included studies, which clearly defined their inclusion criteria that were representative of the general knee OA population, and reported baseline detailed characteristics of the included patients. Some degree of selection bias, the risk of selective loss to follow-up, and the possible presence of confounders in their analysis entailed a moderate to high risk of bias in the other included studies. There was a 96% agreement between the two authors involved in the evaluation of the risk of bias.

### 3.4. Best Evidence Synthesis of the Predictors of Pain Trajectories

Patient-related factors identified as predictors of a severe or progressing pain trajectory, with a strong level of evidence, were low education level and presence of comorbidities (Table 3). In particular, patients without a college degree (primary or secondary school) or patients with one or more comorbidities (in one article they were specified as: asthma, chronic sinusitis, cardiovascular disease, hypertension, gastric ulcer, gallstones, liver disease, renal disease, diabetes, thyroid gland disease, epilepsy, cancer, severe skin disease, and other chronic musculoskeletal diseases) were associated with trajectories characterized by greater pain. The literature showed strong evidence that age, alcohol consumption, and smoking were not predictors of pain evolution. A moderate level of evidence supported depression as a predictor of a severe or progressing pain trajectory, whereas pain coping strategies (such as pain transformation, distraction, reducing demand, retreating, and resting), and the use of medications were shown not to be related to pain evolution. Conflicting findings were reported for BMI, sex, ethnicity, pain at the ipsilateral hip, and worrying as a coping strategy. The evidence was limited for all other factors, since they were reported in only one study.

Regarding joint-related factors, the current literature did not present predictors or related factors that could be identified with a strong or moderate level of evidence (Table 4). Conflicting evidence was found for baseline knee OA (Kellgren–Lawrence grade), and baseline pain and function (Western Ontario and McMaster Universities Arthritis Index—WOMAC). The evidence was limited for all the other factors since they were reported in only one study.

Only one study was focused on short-term pain trajectories, thus providing only limited evidence. A long-lasting disease (>5 years), a higher incidence of flare-ups, and a long-lasting morning stiffness (>15 min) were related to an unstable pain trajectory. No correlations were found for age, gender, BMI, professional activities, anxiety, depression, pain intensity, joint stiffness, knee function, and neuropathic characteristics of pain.

## 4. Discussion

The main finding of this systematic review is that approximately 85% of the patients with early/moderate knee OA had a constant level of pain over time during a mid-term follow-up, whereas 7% of the patients experienced a worsening pain trajectory and 8% of them reported a reduction of pain.

The identification of different pain trajectories supports the hypothesis that several knee OA phenotypes exist [34], with a minority of them associated with a rapidly invalidating disease. In particular, these results showed that, among the vast majority of patients with knee OA having a constant level of pain over the years, only 14% of them suffered from a constant moderate/severe pain. Moreover, 7% of the subjects presented a worsening in pain trajectory in the mid-term follow-up. The understanding of the predictive factors for this subpopulation of patients could help to properly define a prognosis and to provide the most suitable treatment approach for the affected patients. In fact, considering the steady pain trajectories of early/moderate knee OA in the mid-term follow-up, it seems legitimate to prefer a “wait and see” approach (a percentage of patients even showed an improvement at 5 years) as a first-line treatment for most of the patients or, at most, a non-invasive approach that could alleviate patient symptoms. On the other hand, a more aggressive approach should be considered for patients with severe or potentially worsening pain. The timing of TKA, which represents the end-stage treatment of knee OA, is a debated aspect in the field of joint replacement surgery [35,36,37], especially in the young population, where postponing metal resurfacing could be useful to limit possible severe complications and unwanted outcomes [38,39]. The results of this review highlight that a correct characterization of patients could be extremely helpful to select the best approach to manage knee OA. The characterization of patients’ trajectories, distinguishing those with stable, improving, or worsening trajectories, could also be paramount to properly plan OA studies, by ensuring a balanced stratification of these patients and the correct interpretation of study results. These baseline characteristics could be used to choose more specific inclusion criteria or to evaluate sub-groups. Moreover, treatment response according to the different pain trajectories could be evaluated, to better understand how much and for which kind of patient a new therapy would work.

A best evidence synthesis was performed in order to characterize patients who have a higher risk of presenting a severe or worsening pain trajectory. Interestingly, only patient-related factors such as low education level, high number of comorbidities, and depression were identified, with a strong to moderate level of evidence, as predictors of a worse pain trajectory. Joint-related factors, such as baseline function and radiographic severity, were reported with conflicting evidence, underlying the unclear relation between bone deformities and symptoms [40,41]. Therefore, great attention should be given to patient characteristics in order to identify those at high-risk and treat them accordingly. Moreover, depression is a modifiable risk factor that can, and should, be addressed to limit the progression of symptoms and, possibly, improve the quality of life of OA patients [42]. The identification of a low educational level as a risk factor poses questions on potentially modifiable education-related and social factors that could influence the evolution of pain [43]. Among these, diet has been documented, although with limited evidence, to have an influence on pain progression in at least one study, with a protective role of a higher daily fiber intake and an opposite role for meat and dairy products. On the other hand, lifestyle-related factors, which have been frequently related to the educational level, such as alcohol consumption and smoking, were not predictive of pain evolution, with a strong level of evidence [44,45]. Unfortunately, the influence of some predictors was analyzed only in few studies, and other factors that may affect pain evolution, such as the presence of pain sensitization [46], the anatomy, and the muscular tropism of the lower limb [47,48], have not been tested. The literature also still lacks information on many other potentially important aspects. New trials on large cohorts of patients with knee OA should focus on these variables to provide a complete characterization of patients at high risk of developing a severe or worsening pain trajectory.

In this light, this systematic review underlined the usefulness of methods that allow to analyze information about inter-individual differences in the evolution of symptoms over time. The heterogeneous pain progression patterns identified by this study suggest that describing the entire population of knee OA using a single trajectory estimate oversimplifies the complex progression patterns of this disease. Latent growth modeling approaches, such as latent class growth analysis and growth mixture modeling, have been extensively used in other fields for identifying homogeneous subpopulations within the larger heterogeneous population and for the identification of meaningful groups or classes of individuals [49,50,51]. This method is feasible and should be increasingly applied for the population of patients with knee OA, in order to capture the evolution of symptoms in different homogenous sub-groups of patients over the years.

The literature analysis showed an overall focus on the mid-term OA progression, with only one study specifically focusing on the short-term assessment. This study allowed to underline another important aspect. When data were collected on a daily basis, approximately 40% of the patients had an unstable pain trajectory. These results state that, even though in the vast majority of the cases knee OA-related pain is constant in the mid-term follow-up, patients frequently experience unstable pain on a daily basis with frequent severe knee symptoms. In this light, a more frequent assessment method could be extremely important to collect the whole pain experience [52]. In fact, the annual assessment performed in the mid-term follow-up studies could be influenced by the fluctuation of the symptoms documented in the 40% of the subjects, and these fluctuations could bias the symptomatology assessment in patients with knee OA. Unfortunately, only one study was focused on pain trajectories created from a daily-based assessment and this interesting feature could not be further analyzed. This is a limitation of this systematic review.

Overall, the limitations of this study include the low number of published articles on pain trajectories in knee OA. This hindered the possibility to analyze some potential predictors of pain evolution (pain sensitization, pain tolerance, etc.) and led to conflicting findings for other factors. In addition, the multifactorial and subjective nature of pain in OA has to be acknowledged, together with the impossibility of the included studies to take into account these aspects (among all activity level and related pain modifications). New trials should analyze these factors in order to provide a better characterization of the different pain trajectories. Moreover, the data of the included trials were from four large databases that enrolled a relatively small number of patients in developed countries (United States, United Kingdom, Netherlands, France) and thus could only be representative of a part of the knee OA population. Unfortunately, a strong meta-analytic quantitative synthesis was not possible due to the high heterogeneity and the paucity of the included data. Thus, data on the prevalence of the different pain trajectories could be reported only as proportions. Finally, the current review on pain trajectories did not allow to investigate specific threshold levels, which would necessitate dedicated analyses to take into consideration the faceted nature and inter-cultural variability of pain and the impact of this symptom on patient life- and health-related decisions.

Despite the aforementioned limitations, this study allowed to highlight some important features of pain evolution in patients with early/moderate knee OA. Approximately 85% of the subjects had a constant level of pain during a mid-term follow-up, whereas 7% of the patients experienced worsening pain and 8% of them reported a reduction of pain. Among the studied predictors of pain evolution, only patient-related factors such as low education level, high number of comorbidities, and depression were found, with a high to moderate level of evidence, to be predictors of pain evolution in knee OA. No correlation was documented with joint-related factors. This is likely to be related to the limited sensitivity of current methods to characterize OA joints, with broad classifications affected by low accuracy. Future studies should focus on a better characterization of OA patients, both in terms of anatomic features, as well as biological and psycho-social characteristics, in order to identify other key predictors for the evolution of pain trajectories and better manage patients with early/moderate knee OA.

## Figures and Tables

**Table 1:** Inclusion and exclusion criteria used for the article selection process.

Inclusion Criteria	Exclusion Criteria
Studies including patients with symptomatic knee OA	Studies on patients treated with TKA or other surgical and non-surgical treatments
Studies assessing pain evolution over time with pain trajectories in patients without treatment	No information on pain evolution or discrete data for patients with knee OA
Level of evidence I–IV	Expert opinions (level of evidence V), reviews, systematic reviews and meta-analyses
Published in English	Preclinical or ex vivo studies

OA: Osteoarthritis, TKA: Total Knee Arthroplasty.

**Table 2:** Characteristics of the included studies and patients.

Study	Origin of Data	Included Patients	Patients at Follow-Up	Age (Mean ± SD)	Females (%)	BMI (Mean ± SD)	Follow-Up Length	Baseline KL Grade	Pain Score Used
Bastick et al., 2016 [28]	CHECK database	743	705	56 ± 5.1	81	26.5 ± 4.3	5 y	58% grade 0, 42% grade 1	NRS 0–10
Collins et al., 2014 [3]	Osteoarthritis Initiative	1753	1448	62.2 ± 8.9	59	30.1 ± 4.9	6 y	52% grade 2, 34% grade 3, 14% grade 4	WOMAC pain 0–100
Dai et al., 2017 [26]	Osteoarthritis Initiative	1852	1852	61.3 ± 9.1	58	NR	8 y	56% grade 0–1, 44% grade 2–4	WOMAC pain 0–20
Halilaj et al., 2018 [27]	Osteoarthritis Initiative	1243	1243	62.1 ± 8.8	63	29 ± 4.8	6 y	100% grade 0–1	WOMAC pain 0–20
Nicholls et al., 2014 [30]	CAS-K	819	570	64 ± 8	54	29.5 ± 5	6 y	54% grade 0, 9% grade 1, 14% grade 2, 12% grade 3, 10% grade 4	WOMAC pain (0–20)
Trouvin et al., 2018 [31]	French GPs	632	632	67.5 ± 9.4	58	27.9 ± 4.6	28 d	NR	NRS 0–10
Wesseling et al., 2015 [29]	CHECK database	705	705	56 ± 5.1	81	26.5 ± 4.3	5 y	61% grade 0, 39% grade 1	NRS 0–10

BMI: Body Mass Index; d: days; GMM: Growth Mixture Modelling; GP: General Practitioner; LGCA: Latent Growth Class Analysis; NR: not reported; NRS: Numeric Rating Scale; SD: Standard Deviation; WOMAC: Western Ontario and McMaster Universities Osteoarthritis Index; y: years.

**Table 3:** Details of the best evidence synthesis of patient-related predictors of pain trajectories.

Predictor	Number of Studies	Significant Predictor	Non-Significant Predictor	Best Evidence Synthesis
Education level	4	L L M M		Strong
Comorbidities	4	L L M M		Strong
Younger age	5	M	L L M M	Strong
Alcohol	2		L L	Strong
Smoking	2		L L	Strong
Depression	2	M M		Moderate
Pain medication	3		L M M	Moderate
Coping (others)	2		L L	Moderate
Diet fibres	1	H		Limited
Physical activity	1		M	Limited
Diet: meat/diary	1		M	Limited
Supplementary Vitamins	1		L	Limited
BMI	5	L L M	M M	Conflicting
Female sex	5	M M	L M M	Conflicting
Pain ipsilateral hip	2	L	L	Conflicting
Black ethnicity	2	M	L	Conflicting
Coping (worrying)	2	L	L	Conflicting

BMI: Body Mass Index; H: High risk of bias; L: Low risk of bias; M: Moderate risk of bias.

**Table 4:** Details of the best evidence synthesis of knee osteoarthritis (OA)-related predictors of pain trajectories.

Predictor	Number of Studies	Significant Predictor	Non-Significant Predictor	Best Evidence Synthesis
Knee tenderness	1	L		Limited
Joint space width	1	M		Limited
Knee warmth	1		L	Limited
Bony enlargement	1		L	Limited
Crepitus	1		L	Limited
Positive re-fill test	1		L	Limited
ROM flexion	1		L	Limited
ROM extension	1		L	Limited
Pain in motion	1		L	Limited
Bouchard	1		L	Limited
Heberden	1		L	Limited
ESR	1		L	Limited
Knee alignment	1		M	Limited
Problems duration	1		M	Limited
Baseline pain	1		L	Limited
WOMAC, stiffness	1		L	Limited
K-L	4	M M	L L	Conflicting
WOMAC, pain	2	H	L	Conflicting
WOMAC, function	2	H	L	Conflicting

ESR: Erythrocyte Sedimentation Rate; H: High risk of bias; K-L: Kellgren–Lawrence; L: Low risk of bias; M: Moderate risk of bias; ROM: Range of Motion.

## Supplementary Materials

The following are available online at https://www.mdpi.com/2077-0383/9/9/2828/s1, Figure S1: Forest plot of the meta-analysis of prevalence for the patients with a constant pain trajectory, Figure S2: Forest plot of the meta-analysis of prevalence for the patients with a constant minimal pain trajectory, Figure S3: Forest plot of the meta-analysis of prevalence for the patients with a constant mild pain trajectory, Figure S4: Forest plot of the meta-analysis of prevalence for the patients with a constant moderate pain trajectory, Figure S5: Forest plot of the meta-analysis of prevalence for the patients with a constant severe pain trajectory, Figure S6: Forest plot of the meta-analysis of prevalence for the patients with an increasing pain trajectory, Figure S7: Forest plot of the meta-analysis of prevalence for the patients with an increasing mild pain trajectory, Figure S8: Forest plot of the meta-analysis of prevalence for the patients with an increasing moderate pain trajectory, Figure S9: Forest plot of the meta-analysis of prevalence for the patients with an increasing severe pain trajectory, Figure S10: Forest plot of the meta-analysis of prevalence for the patients with a decreasing pain trajectory, Figure S11: Forest plot of the meta-analysis of prevalence for the patients with a decreasing mild pain trajectory, Figure S12: Forest plot of the meta-analysis of prevalence for the patients with a decreasing moderate pain trajectory, Figure S13: Forest plot of the meta-analysis of prevalence for the patients with a decreasing severe pain trajectory, Table S1: PRISMA 2009 Checklist.

## References

[1] Vos T., Flaxman A.D., Naghavi M., Lozano R., Michaud C., Ezzati M., Shibuya K., Salomon J.A., Abdalla S., Aboyans V. (2012). Years lived with disability (YLDs) for 1160 sequelae of 289 diseases and injuries 1990–2010: A systematic analysis for the Global Burden of Disease Study 2010. Lancet.

[2] Bartlett S.J., Ling S.M., Mayo N.E., Scott S.C., Bingham C.O. (2011). Identifying common trajectories of joint space narrowing over two years in knee osteoarthritis. Arthritis Care Res. (Hoboken).

[3] Collins J.E., Katz J.N., Dervan E.E., Losina E. (2014). Trajectories and risk profiles of pain in persons with radiographic, symptomatic knee osteoarthritis: Data from the osteoarthritis initiative. Osteoarthr. Cartil..

[4] Nguyen U.S., Zhang Y., Zhu Y., Niu J., Zhang B., Felson D.T. (2011). Increasing prevalence of knee pain and symptomatic knee osteoarthritis: Survey and cohort data. Ann. Intern. Med..

[5] Peat G., McCarney R., Croft P. (2001). Knee pain and osteoarthritis in older adults: A review of community burden and current use of primary health care. Ann. Rheum. Dis..

[6] Zhang W., Robertson J., Jones A.C., Dieppe P.A., Doherty M. (2008). The placebo effect and its determinants in osteoarthritis: Meta-analysis of randomised controlled trials. Ann. Rheum. Dis..

[7] Filardo G., Kon E., Longo U.G., Madry H., Marchettini P., Marmotti A., Van Assche D., Zanon G., Peretti G.M. (2016). Non-surgical treatments for the management of early osteoarthritis. Knee Surg. Sports Traumatol. Arthrosc..

[8] Filardo G., Previtali D., Napoli F., Candrian C., Zaffagnini S., Grassi A. (2020). PRP Injections for the Treatment of Knee Osteoarthritis: A Meta-Analysis of Randomized Controlled Trials. Cartilage.

[9] De Girolamo L., Kon E., Filardo G., Marmotti A.G., Soler F., Peretti G.M., Vannini F., Madry H., Chubinskaya S. (2016). Regenerative approaches for the treatment of early OA. Knee Surg. Sports Traumatol. Arthrosc..

[10] Vannini F., Spalding T., Andriolo L., Berruto M., Denti M., Espregueira-Mendes J., Menetrey J., Peretti G.M., Seil R., Filardo G. (2016). Sport and early osteoarthritis: The role of sport in aetiology, progression and treatment of knee osteoarthritis. Knee Surg. Sports Traumatol. Arthrosc..

[11] Felson D., Niu J., Sack B., Aliabadi P., McCullough C., Nevitt M.C. (2013). Progression of osteoarthritis as a state of inertia. Ann. Rheum. Dis..

[12] Croft P., Porcheret M., Peat G. (2011). Managing osteoarthritis in primary care: The GP as public health physician and surgical gatekeeper. Br. J. Gen. Pract..

[13] Losina E., Weinstein A.M., Reichmann W.M., Burbine S.A., Solomon D.H., Daigle M.E., Rome B.N., Chen S.P., Hunter D.J., Suter L.G. (2013). Lifetime risk and age at diagnosis of symptomatic knee osteoarthritis in the US. Arthritis Care Res. (Hoboken).

[14] Li C.S., Karlsson J., Winemaker M., Sancheti P., Bhandari M. (2014). Orthopedic surgeons feel that there is a treatment gap in management of early OA: International survey. Knee Surg. Sports Traumatol. Arthrosc..

[15] Arias-Vazquez P.I., Tovilla-Zarate C.A., Legorreta-Ramirez B.G., Burad Fonz W., Magana-Ricardez D., Gonzalez-Castro T.B., Juarez-Rojop I.E., Lopez-Narvaez M.L. (2019). Prolotherapy for knee osteoarthritis using hypertonic dextrose vs other interventional treatments: Systematic review of clinical trials. Adv. Rheumatol..

[16] Lohmander L.S., Roos E.M. (2019). Disease modification in OA—Will we ever get there?. Nat. Rev. Rheumatol..

[17] Nevitt M., Felson D., Lester G. (2006). The osteoarthritis initiative: Protocol for the Cohort Study. OARSI J..

[18] Jung T., Wickrama K.A. (2008). An introduction to latent class growth analysis and growth mixture modeling. Soc. Personal. Psychol. Compass.

[19] Losina E., Collins J.E. (2016). Forecasting the future pain in hip OA: Can we rely on pain trajectories?. Osteoarthr. Cartil..

[20] Moher D., Liberati A., Tetzlaff J., Altman D.G. (2009). Preferred reporting items for systematic reviews and meta-analyses: The PRISMA statement. PLoS Med..

[21] Higgins J.P.T., Green S. (2011). Chapter 7: Selecting studies and collecting data. Cochrane Handbook for Systematic Reviews of Interventions.

[22] Ioannidis J.P., Patsopoulos N.A., Rothstein H.R. (2008). Reasons or excuses for avoiding meta-analysis in forest plots. BMJ.

[23] Slavin R.E. (1995). Best evidence synthesis: An intelligent alternative to meta-analysis. J. Clin. Epidemiol..

[24] van Tulder M., Furlan A., Bombardier C., Bouter L. (2003). Updated method guidelines for systematic reviews in the cochrane collaboration back review group. Spine.

[25] Eijgenraam S.M., Reijman M., Bierma-Zeinstra S.M.A., van Yperen D.T., Meuffels D.E. (2018). Can we predict the clinical outcome of arthroscopic partial meniscectomy? A systematic review. Br. J. Sports Med..

[26] Dai Z., Lu N., Niu J., Felson D.T., Zhang Y. (2017). Dietary Fiber Intake in Relation to Knee Pain Trajectory. Arthritis Care Res. (Hoboken).

[27] Halilaj E., Le Y., Hicks J.L., Hastie T.J., Delp S.L. (2018). Modeling and predicting osteoarthritis progression: Data from the osteoarthritis initiative. Osteoarthr. Cartil..

[28] Bastick A.N., Wesseling J., Damen J., Verkleij S.P., Emans P.J., Bindels P.J., Bierma-Zeinstra S.M. (2016). Defining knee pain trajectories in early symptomatic knee osteoarthritis in primary care: 5-year results from a nationwide prospective cohort study (CHECK). Br. J. Gen. Pract..

[29] Wesseling J., Bastick A.N., Ten Wolde S., Kloppenburg M., Lafeber F.P., Bierma-Zeinstra S.M., Bijlsma J.W. (2015). Identifying Trajectories of Pain Severity in Early Symptomatic Knee Osteoarthritis: A 5-year Followup of the Cohort Hip and Cohort Knee (CHECK) Study. J. Rheumatol..

[30] Nicholls E., Thomas E., van der Windt D.A., Croft P.R., Peat G. (2014). Pain trajectory groups in persons with, or at high risk of, knee osteoarthritis: Findings from the Knee Clinical Assessment Study and the Osteoarthritis Initiative. Osteoarthr. Cartil..

[31] Trouvin A.P., Marty M., Goupille P., Perrot S. (2019). Determinants of daily pain trajectories and relationship with pain acceptability in hip and knee osteoarthritis. A national prospective cohort study on 886 patients. Jt. Bone Spine.

[32] Luyten F.P., Denti M., Filardo G., Kon E., Engebretsen L. (2012). Definition and classification of early osteoarthritis of the knee. Knee Surg. Sports Traumatol. Arthrosc..

[33] Madry H., Kon E., Condello V., Peretti G.M., Steinwachs M., Seil R., Berruto M., Engebretsen L., Filardo G., Angele P. (2016). Early osteoarthritis of the knee. Knee Surg. Sports Traumatol. Arthrosc..

[34] Deveza L.A., Melo L., Yamato T.P., Mills K., Ravi V., Hunter D.J. (2017). Knee osteoarthritis phenotypes and their relevance for outcomes: A systematic review. Osteoarthr. Cartil..

[35] Cross W.W., Saleh K.J., Wilt T.J., Kane R.L. (2006). Agreement about indications for total knee arthroplasty. Clin. Orthop. Relat. Res..

[36] Jain N.B., Higgins L.D., Ozumba D., Guller U., Cronin M., Pietrobon R., Katz J.N. (2005). Trends in epidemiology of knee arthroplasty in the United States, 1990–2000. Arthritis Rheum..

[37] Roemer F.W., Kwoh C.K., Hannon M.J., Hunter D.J., Eckstein F., Fujii T., Boudreau R.M., Guermazi A. (2015). What comes first? Multitissue involvement leading to radiographic osteoarthritis: Magnetic resonance imaging-based trajectory analysis over four years in the osteoarthritis initiative. Arthritis Rheumatol..

[38] Scott C.E., Oliver W.M., MacDonald D., Wade F.A., Moran M., Breusch S.J. (2016). Predicting dissatisfaction following total knee arthroplasty in patients under 55 years of age. Bone Jt. J..

[39] Hawker G.A., Badley E.M., Borkhoff C.M., Croxford R., Davis A.M., Dunn S., Gignac M.A., Jaglal S.B., Kreder H.J., Sale J.E. (2013). Which patients are most likely to benefit from total joint arthroplasty?. Arthritis Rheum..

[40] Jones L.D., Bottomley N., Harris K., Jackson W., Price A.J., Beard D.J. (2016). The clinical symptom profile of early radiographic knee arthritis: A pain and function comparison with advanced disease. Knee Surg. Sports Traumatol. Arthrosc..

[41] Baert I.A., Staes F., Truijen S., Mahmoudian A., Noppe N., Vanderschueren G., Luyten F.P., Verschueren S.M. (2014). Weak associations between structural changes on MRI and symptoms, function and muscle strength in relation to knee osteoarthritis. Knee Surg. Sports Traumatol. Arthrosc..

[42] Han H.S., Lee J.Y., Kang S.B., Chang C.B. (2016). The relationship between the presence of depressive symptoms and the severity of self-reported knee pain in the middle aged and elderly. Knee Surg. Sports Traumatol. Arthrosc..

[43] Creamer P., Hochberg M.C. (1998). The relationship between psychosocial variables and pain reporting in osteoarthritis of the knee. Arthritis Care Res..

[44] Moore A.A., Gould R., Reuben D.B., Greendale G.A., Carter M.K., Zhou K., Karlamangla A. (2005). Longitudinal patterns and predictors of alcohol consumption in the United States. Am. J. Public Health.

[45] Giskes K., Kunst A.E., Benach J., Borrell C., Costa G., Dahl E., Dalstra J.A., Federico B., Helmert U., Judge K. (2005). Trends in smoking behaviour between 1985 and 2000 in nine European countries by education. J. Epidemiol. Community Health.

[46] Carlesso L.C., Segal N.A., Frey-Law L., Zhang Y., Na L., Nevitt M., Lewis C.E., Neogi T. (2019). Pain Susceptibility Phenotypes in Those Free of Knee Pain with or at Risk of Knee Osteoarthritis: The Multicenter Osteoarthritis Study. Arthritis Rheumatol..

[47] Hunter D.J., Niu J., Zhang Y., Nevitt M.C., Xu L., Lui L.Y., Yu W., Aliabadi P., Buchanan T.S., Felson D.T. (2005). Knee height, knee pain, and knee osteoarthritis: The Beijing Osteoarthritis Study. Arthritis Rheum..

[48] Lee J.Y., Han K., McAlindon T.E., Park Y.G., Park S.H. (2018). Lower leg muscle mass relates to knee pain in patients with knee osteoarthritis. Int. J. Rheum. Dis..

[49] Baron E., Bass J., Murray S.M., Schneider M., Lund C. (2017). A systematic review of growth curve mixture modelling literature investigating trajectories of perinatal depressive symptoms and associated risk factors. J. Affect. Disord..

[50] Berlin K.S., Parra G.R., Williams N.A. (2014). An introduction to latent variable mixture modeling (part 2): Longitudinal latent class growth analysis and growth mixture models. J. Pediatr. Psychol..

[51] Musliner K.L., Munk-Olsen T., Eaton W.W., Zandi P.P. (2016). Heterogeneity in long-term trajectories of depressive symptoms: Patterns, predictors and outcomes. J. Affect. Disord..

[52] Stone A.A., Schwartz J.E., Broderick J.E., Shiffman S.S. (2005). Variability of momentary pain predicts recall of weekly pain: A consequence of the peak (or salience) memory heuristic. Pers. Soc. Psychol. Bull..

